# A Transient Receptor Potential-like Calcium Ion Channel in the Filamentous Fungus *Aspergillus nidulans*

**DOI:** 10.3390/jof7110920

**Published:** 2021-10-28

**Authors:** Hongchen Wang, Qiuyi Chen, Shizhu Zhang, Ling Lu

**Affiliations:** Jiangsu Key Laboratory for Microbes and Functional Genomics, Jiangsu Engineering and Technology Research Center for Microbiology, College of Life Sciences, Nanjing Normal University, Nanjing 210023, China; 191201011@njnu.edu.cn (H.W.); qiuyi.chen@genscript.com (Q.C.)

**Keywords:** Transient Receptor Potential (TRP), calcium, cell wall, *Aspergillus nidulans*

## Abstract

Transient Receptor Potential (TRP) proteins constitute a superfamily that encodes transmembrane ion channels with highly diverse permeation and gating properties. Filamentous fungi possess putative TRP channel-encoded genes, but their functions remain elusive. Here, we report that a putative TRP-like calcium channel, *trpR,* in the filamentous fungus *Aspergillus nidulans*, performs important roles in conidiation and in adapting to cell wall disruption reagents in a high temperature-induced defect-dependent manner, especially under a calcium-limited culture condition. The genetic and functional relationship between TrpR and the previously identified high-affinity calcium channels CchA/MidA indicates that TrpR has an opposite response to CchA/MidA when reacting to cell wall disruption reagents and in regulating calcium transients. However, a considerable addition of calcium can rescue all the defects that occur in TrpR and CchA/MidA, meaning that calcium is able to bypass the necessary requirement. Nevertheless, the colocalization at the membrane of the Golgi for TrpR and the P-type Golgi Ca^2+^ ATPase PmrA suggests two channels that may work as ion transporters, transferring Ca^2+^ from the cytosol into the Golgi apparatus and maintaining cellular calcium homeostasis. Therefore, combined with data for the *trpR* deletion mutant revealing abnormal cell wall structures, TrpR works as a Golgi membrane calcium ion channel that involves cell wall integration.

## 1. Introduction

Transient receptor potential (TRP) proteins were first described in *Drosophila* [1], and the presence of their superfamilies, that possess highly diverse permeation and gating properties, has been reported in most eukaryotes [2,3,4]. To date, around 30 proteins have been reported to belong to the TRP superfamily and possess some similar structural characteristics. Based on sequence homology, these proteins have been subdivided into eight major branches and are divided into three broad groups: group one includes TRPC, TRPA, TRPM, TRPN, TRPV, group two includes TRPP and TRPML, and group three is the TRPY subfamily which currently only has one member, TRPY1 (or Yvc1). Furthermore, members of group one and two are all found in the metazoans but TRPY1(TRPY subfamily) was identified in fungi which putatively evolved from metazoans to fungi and resulted in a divergence [5]. However, TRP proteins seem to be absent in archaea, bacteria or higher plants [2,6,7].

In mammals, most TRP members are permeable for both Ca^2+^ or Mg^2+^, and some are even highly permeable relative to the monovalent cations [2]. TRP channels are best recognized as sensors for environmental irritants, causing somatosensory modalities, such as pain, cold, itching, and other protective responses [3,6]. It has been found that TRP mutations are linked to human diseases, indicating the physiological importance of TRP proteins [4,7]. The mammalian TRP protein forms a tetramer, where each polypeptide consists of six transmembrane domains (TMDs) and the putative ion conducting pore is located between the fifth and sixth TMDs [7,8].

Yeast fungal homologs share a similar predicted membrane topology to that in mammals, and some of the regulatory sites are located in the cytosolic C-terminal region [9,10,11]. In addition, the TRP channel subunits in fungi also contain at least six predicted TMDs, suggesting that their topologies are similar to that of human TRP channel subunits [7,8].

In the budding yeast *Saccharomyces cerevisiae*, TRPY1 has been identified for its function as a vacuolar channel responsible for Ca^2+^ release in response to osmotic stress, and its activation and inhibition are modulated by calcium and lipid, respectively [12]. In the fission yeast *Schizosaccharomyces pombe*, three TRP-like ion channel encoding genes have been reported: *pkd2* (SPAC1F7.03), *trp1322* (SPCC1322.03), and *trp663* (SPCC663.14c) [13,14]. The functions of Pkd2, playing an important role in cell wall synthesis, membrane protein trafficking and regulating cell separation during cytokinesis have been reported [15,16]. In addition, Pkd2 and Trp1322 are both Ca^2+^-permeable and can mediate the cytoplasmic Ca^2+^ response, while no detectable functions of Trp663 have been found [13]. Interestingly, homologs of Pkd2 in some fungi have been proposed to be members of the FLC family, responsible for flavin adenine dinucleotide (FAD) transporting, but they also have calcium channel activity [17].

Calcium ion (Ca^2+^) is a ubiquitous intracellular second messenger and performs an important role in regulating a wide range of processes, including cell morphogenesis, cell cycle progression, stress responses and virulence for all eukaryotic cells [18]. In the resting state, the concentration of cytosolic free Ca^2+^ is maintained at a very low level, for which the majority of intracellular Ca^2+^ is stored in intracellular storage organelles such as the endoplasmic reticulum, Golgi apparatus, vacuole, and mitochondria [19]. Upon stimulation, Ca^2+^ enters from the extracellular environment and is released from the intracellular Ca^2+^ pools to the cytosol, rapidly and simultaneously. Transient changes in cytoplasmic Ca^2+^ levels can activate various Ca^2+^-sensing proteins, and various downstream signal transmission pathways are induced to process the abovementioned stimulation. Finally, Ca^2+^ is reset to the resting level stage for the commencement of the next cycle. Therefore, calcium homeostasis in the cell is maintained by a variety of transporters, channels, and pumps located in the plasma membrane or stored in intracellular storage organelles. In fungi, two different calcium uptake systems have been identified: the high-affinity Ca^2+^ influx system (HACS) and the low-affinity calcium influx system (LACS) [20]. Under low-calcium conditions, the main components of the HACS (Cch1/Mid1 in yeast or CchA/MidA in *Aspergillus* spp.) must function in calcium uptake from the extracellular media [21,22]. Losses of *cch1*/*mid1* (CchA/MidA) consistently result in defects in vegetative growth, sexual and/or asexual development, and pathogenicity for some species [21]. Additionally, fungi possess a range of calcium P-type ATPases and calcium transporters that are responsible for pumping Ca^2+^ against ion gradients at the expense of energy derived from ATP and maintaining this gradient differential between intracellular Ca^2+^ pools and the cytoplasm [23]. In yeast, the Golgi Ca^2+^ pump Pmr1 has been identified, and its ortholog in *Aspergillus nidulans* is PmrA, found to be associated with their growth, morphogenesis and the cell wall integrity [24,25].

In this study, according to a bioinformatic BLAST analysis for the TRP Pfam (PF06011) in the genome of the filamentous fungi *Aspergillus nidulans*, we identified that the Golgi-localized TRP-like protein TrpR performs an important function in cellular calcium equilibrium and cell wall integration in *A. nidulans*.

## 2. Materials and Methods

### 2.1. Strains, Media, and Growth Conditions

The *A. nidulans* strains used in this study have been summarized and listed (see Table 1). *A. nidulans* strains were grown on rich media YAG or YUU (YAG: 2% glucose, 1 mL·L^−1^ trace elements, 0.5% yeast extract, 2% agar (for solid). YUU: YAG + 5 mM uridine + 10 mM uracil). Experiments were performed in a minimal amount of medium PDR or PDRUU (PDR: 2% glucose, 50 mL·L^−1^ salts, 1 mL·L^−1^ 1000 × trace elements solution, 2.5 μM pyridoxine, 6.6 μM riboflavin. PDRUU: PDR + 5 mM uridine + 10 mM uracil, 1000 × trace elements solution and 20 × Salts solution were prepared according to the formulation provided in the literature [26]. The recipe of the 1000 × trace elements solution is as follows: 77 mM ZnSO_4_·7H_2_O, 177 mM H_3_BO_3_, 31 mM MnCl_2_·4H_2_O, 18 mM FeSO_4_·7H_2_O, 6.7 mM CoCl_2_·6H_2_O, 10 mM CuSO_4_, 1 mM (NH_4_)_6_Mo_7_O_24_·4H_2_O, 132 mM EDTA. The recipe of 20 × salts solution is followed: NaNO_3_ 1.4 M, KCL 0.14 M, KH_2_PO_4_ 0.22 M, MgSO_4_·7H_2_O 42 mM.), maintained at 37 °C. The Conidia were harvested on YAG plates using sterile H_2_O and placed, for long-term storage, in 50% glycerol at −80 °C. Additional instructions will be provided if special cultivation conditions are required.

### 2.2. Genetic Mutant Strain Construction

To construct the deletion mutant strains in this study, homologous recombination was used [27]. All primers used to design constructs are listed in Table 2. Fungal transformation was also performed as previously described [28]. For construction of Δ*trpR*, we used *pyrG* as a selectable nutritional marker and amplified the 5′ and 3′ flanking regions of the *trpR* open reading frame (ORF) from *A. nidulans* TN02A7 genomic DNA with primer pairs trpR P1/P3 and trpR P4/P6, respectively. Finally, combining these three fragments was achieved through fusion PCR using the primer pair trpR P2/P5. The PCR product was transformed into the recipient wild-type strain.

To construct the *trpR* complemented strain, we amplified the *trpR* gene including the promoter, ORF, and terminator, with the primer pairs trpR up/down from *A. nidulans* gDNA. We cloned the *trpR* gene into the plasmid pQa-pyroA, which contains a 1.7 kb *pyroA* fragment as a selectable marker. The new plasmid was transformed into the recipient Δ*trpR* strain to generate the *trpR^c^* strain.

To generate the TrpR-GFP strain, we followed the same principle used for building deletion mutant strains. In summary, the 5′ and 3′ flanking regions of the *trpR* stop codon were amplified from strain TN02A7 using primer pairs trpR-gfp P1/P3 and trpR-gfp P4/P6, respectively. The *gfp*-*pyrG* fragment was amplified from the plasmid pFNO3 using primers gfp up and pyrG down. The *trpR-gfp-pyrG* fusion PCR product (using primer pairs trpR-gfp P2/P5) was transformed into the recipient strain TN02A7. The TrpR-GFP mRFP-PH^OSBP^ and TrpR-GFP RFP-PmrA strains were constructed by transforming the relative gene fragments into the recipient TrpR-GFP strain. The transformants were verified using diagnostic PCR and microscopic testing.

The Δ*pmrA gpd-trpR* and Δ*trpR gpd-pmrA* strains were constructed using a similar strategy. To summarize, the *gpd* promoter was amplified with primer pairs gpd up/down, and the *trpR* gene fragment was amplified with primers gpd-trpR up/down. These two fragments were combined using primers gpd up and gpd-trpR down. Finally, the above fusion product was cloned into the plasmid pQa-pyroA to create a new plasmid. We transformed it into the recipient Δ*pmrA* strain to obtain the Δ*pmrA gpd-trpR* strain. Similar approaches were applied for the construction of the Δ*trpR gpd-pmrA* strain. And the transformers were verified using qRT-PCR assays.

The strains that expressed codon-optimized aequorin were constructed by co-transforming the plasmid pAEQ containing aequorin and the selective markers *pyroA* or *riboB* genes into the indicated mutants [29]. We screened the transformers for aequorin expression using diagnostic PCR assays and selected the suitable aequorin expressing strains for further purification.

The double deletion strain was generated by crossing two relative single deletion strains as previously described [30]. In brief, we inoculated two parent strains on a plate for 2 days at 37 °C. Then, the mixed-grown mycelia were transferred to a screening medium supplemented with 66 μM riboflavin and incubated for 15 days at 30 °C until cleistothecia became visible. A grain of cleistothecium was lifted, and the mycelia around it were cleaned, after which a cleaned cleistothecium was transferred to a 1.5-mL centrifuge tube. Then, 1 mL of sterilized H_2_O was added, and the solution was mixed well. Finally, a 10-μL suspension of ascospores was transferred to the rich YUU medium for culturing. The progenies were screened based on their phenotypes.

### 2.3. Phylogenetic Analysis

All of the amino acid sequences were obtained from the fungal databases FungiDB (http://fungidb.org/fungidb, accessed on 26 October 2021) and NCBI (https://www.ncbi.nlm.nih.gov, accessed on 26 October 2021). The Hidden Markov Model (HMM) profile for the TRP Pfam (PF06011) was downloaded from the protein family database (http://pfam.xfam.org, accessed on 26 October 2021) and was used to identify the putative TRP channel genes from the A. nidulans genome with HMMER 3.0 (http://hmmer.janelia.org/, accessed on 31 July 2020) [31]. The phylogenetic tree was constructed via MEGA 7 software, using the neighbor-joining method and a bootstrap test with 1000 iterations.

### 2.4. Plate

To analyze the impact of thermal treatment on conidiation, wild-type and relevant strains were cultured at 30, 37 and 42 °C. To obtain the impact of cell wall stress, we tested the condition of fungal growth on a medium supplemented with the following agents: calcofluor white (CFW), congo red (CR), and caspofungin (CAS) (Sigma-Aldrich, St. Louis, MO, USA). In order to test the restorative effect of calcium on fungal growth, 50 mM of CaCl_2_ was added to the medium. The operation process was as follows: 2.5 μL of the conidia (1 × 10^6^ conidia·mL^−1^) of the indicated strains were spotted onto relevant media and cultured for 2.5 days. Finally, the colony diameter was measured, and the total spore quantity of all strains was counted. At least three replicates were performed for each experiment.

### 2.5. Microscopic Observation and Image Processing

In order to observe the hyphal growth, approximately 1 × 10^4^ conidia of the relevant strains were incubated in a 1 mL liquid PDR or PDRUU medium and cultured in a petri dish containing a coverslip, at 37 °C for approximately 10 h and observed under a microscope. For the localization of the TrpR-GFP protein, we incubated the TrpR-GFP strain in PDR medium. After 10 h, we removed the medium and washed the mycelia three times with phosphate-buffered saline. Then, the mycelia were fixed with 4% paraformaldehyde (Polysciences, Warrington, PA, USA) for 40 min at room temperature in the dark. The paraformaldehyde was cleaned and visualized under a fluorescence microscope using a 63× objective oil lens. FM4–64 (Sigma-Aldrich, St. Louis, MO, USA) staining was conducted on the ice in accordance with the protocol manual. All the images were captured with a Zeiss Axio imager A1 microscope (Carl Zeiss, Jena, Germany).

### 2.6. RNA Isolation and Quantitative RT-PCR Assays

For RNA isolation, 1 × 10^8^ fresh conidia from related strains were inoculated in 100 mL of a liquid PDR or PDRUU medium at 37 °C for 16 h, and then the mycelia were harvested and frozen in liquid nitrogen. RNA was extracted using liquid nitrogen and an RNAzol RT column kit (Sangon Biotech no. B511631-0100). Both the reverse transcription-PCR and qRT-PCR analyses were performed using HiScript II Reverse Transcriptase (Vazyme catalog no. R201-01) and SYBR Premix Ex Taq (TaKaRa catalog no. DRR041A), respectively. The specific operations complied with the instructions of the protocol manual, and the transcription levels were calculated according to the comparative threshold cycle (Δ^CT^) method [27].

### 2.7. Cytoplasmic Ca^2+^ Measurement

Cytoplasmic Ca^2+^ determination was performed as previously described [19,32]. We briefly transformed the pAEQ vector harboring the codon-optimized aequorin gene into the indicated strains. The strains expressing the aequorin gene were cultured in a PDRUU medium, adjusted to 1 × 10^7^ spores·ml^−1^, and then distributed into wells in a 96-well microdroplet plate at 100 μL per well. Each strain was inoculated with 8 parallel replicates. After their incubation for 18 h at 37 °C, the medium was removed, and the mycelia were rinsed twice with PGM (50 mM glucose, 1 mM MgCl_2_ and 20 mM PIPES (pH 6.7)). The aequorin was reconstituted by incubating mycelia in 100 μL 25 μM·ml^−1^ coelenterazine (Sigma-Aldrich, St. Louis, MO, USA) diluted with PGM for 4 h at 4 °C in the dark. After reconstitution, the mycelia were washed twice with PGM, and the plate was placed at room temperature for 1 h. The luminescence, excited by 10 mM CaCl_2_,was measured with an LB 96P Microlumat luminometer (Berthold Technologies, Bad Wildbad, Germany) and the active aequorin was completely discharged using a discharge buffer (containing 20% (vol/vol) ethanol and 3 M CaCl_2_). Finally, the relative light unit (RLU) values were converted into [Ca^2+^]c concentrations through the use of the following calibration formula: pCa = 0.332588 (−log k) + 5.5593, where k is luminescence (in RLU) s^−1^/total luminescence (in RLU) [29].

### 2.8. Statistical Analysis

Data were provided by means of ± SD. The statistical significance was estimated using either *t*-tests or multiple *t*-tests. The *p*-values of less than 0.05 were considered statistically significant.

## 3. Results

### 3.1. Phylogenetic Evolution and Diversification of TrpR Homologs in Selected Eukaryotic Species

**Figure 1:**
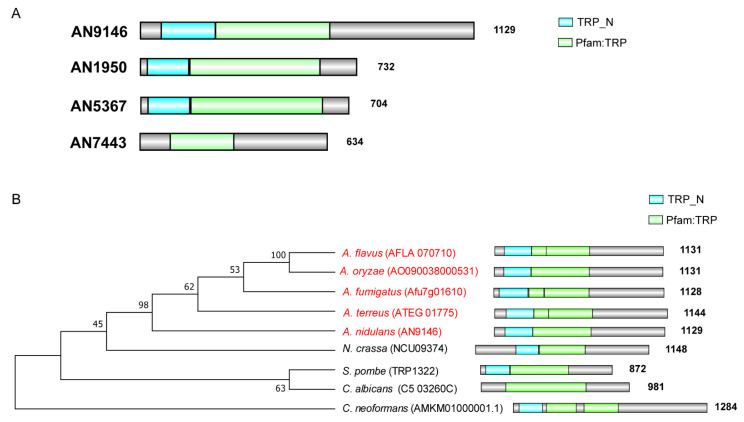
Members of fungal TRPs have similarly conserved domains. (**A**) The illustration shows the 4 TRP domain-containing candidates for filamentous fungi (*A. nidulans*). (**B**) Phylogenetic relationships of the homologs of TrpR. This tree includes selected organisms representing major fungal groups according to the labels. Red labels indicate *Aspergillus* spp. The tree was reconstructed using the neighbor-joining method.

To study the phylogenetic relationship of all proteins harboring the TRP Pfam in *A. nidulans* database, a Hidden Markov Model profiling (hmmscan) was performed against the *A. nidulans* protein database using the TRP Pfam (PF06011) as a searching criterion [31]. As shown in Figure 1A, there are 4 putative TRP-like channels in *A. nidulans* which contain the TRP Pfam domain in their protein sequence. Among them, the gene numbered AN9146 is matched with a putative homolog of *S. pombe trp1322* which had been reported for its functions of mediating the cytoplasmic Ca^2+^ in yeasts. We then focused on the functions of AN9146 which we referred to as a *trpR.*

To further explore the conservative property of TrpR homologs, we performed an evolutionary analysis using the TrpR’s total protein sequence to perform a BLAST search in selected eukaryotes, as presented in Figure 1B. As a result, all the selected ascomycete homologs present a relatively close relationship between them (Figure 1B). Homologs in *Aspergillus* spp are very closely related, such that *A. nidulans* demonstrated, approximately, a 65–90% amino acid sequence, identifying with other species of *Aspergillus*. In comparison, the TrpR in *A. nidulans* (AN9146) has a 43% identification with *Neurospora crassa* whereas TrpR only presented 25% amino acid sequence identity with *S. pombe,* 24% with *Cryptococcus neoformans*, 22% with *Candida albicans*. Further phylogenetic analyses indicate that all selected ascomycete homologs possess signatures that are typical for the TRP_N domain and TRP Pfam, suggesting that the TrpR homologs are relatively conserved in fungi (Figure 1B).

### 3.2. Dynamic Localization of TrpR-GFP during Different Developmental Stages of A. nidulans

**Figure 2:**
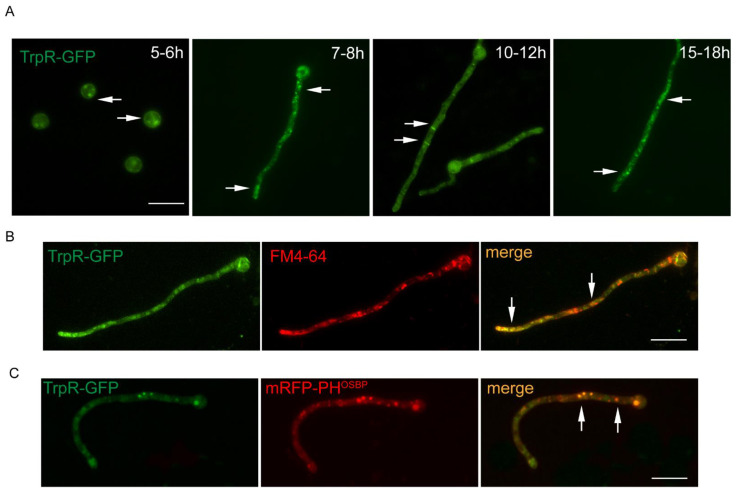
TrpR showed a multiple type of distribution. All panels show epifluorescence microscopy analyses of 6–12 h growing hyphal cells in liquid PDR medium at 37 °C. (**A**) Subcellular localizations of TrpR-GFP in different developmental stages. (**B**) Epifluorescence microscopy showing the subcellular localization of TrpR-GFP with FM4–64 staining, which labels the lipid membrane structure. (**C**) Distribution of TrpR-GFP relative to the late Golgi marker PH^OSBP^. Overlapping positions are indicated with an arrow at the merged picture, Scale bar, 5 μm.

To further study the localization of TrpR, a TrpR-GFP strain with a green fluorescent protein (GFP) tag fused at the C-terminus of TrpR was constructed. We confirmed it to be successful by a diagnostic PCR (see Figure S1A). As shown in Figure 2A, the green fluorescence of the TrpR-GFP fusion protein possesses a multiple-type distribution under the different developmental stages. In swollen conidia, TrpR displayed a weak plasma membrane pattern in the form of a circle along the conidial cell wall, accompanied by deep-green particles of the endomembrane system. In the germlings, TrpR-GFP predominantly accumulated in the apical region of hyphae. In mature hyphae, TrpR-GFP presented septum-type and cytoplasmic-punctuated locations.

To further explore the location of TrpR, we used the lipophilic marker FM4–64, a lipid membrane marker, to stain the TrpR-GFP strain. The results suggest that the majority of the TrpR-GFP foci was stained by FM4–64, especially in the subapical region (see Figure 2B). Additionally, we used the human oxysterol-binding protein PH domain (PH^OSBP^), which is a well-established marker to label late/trans-Golgi compartments, to examine the possible colocalization association of TrpR-GFP with Golgi compartments. As shown in Figure 2C, TrpR exhibited partial puncta colocalization with the late Golgi marker mRFP-PH^OSBP^. Overall, TrpR is an endomembrane protein that is usually located in the membrane of Golgi and vesicle-like membrane structures.

### 3.3. Lack of TrpR Causes Hypersensitivity to Thermal Stresses and Cell Wall Destruction Reagents

**Figure 3:**
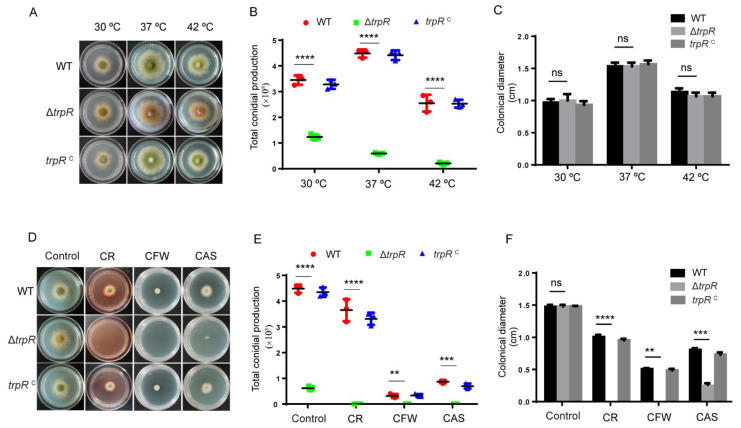
TrpR is important for adapting to thermal and cell wall stress agents. (**A**) Colony morphology of the Δ*trpR* and reference strains on the solid PDRUU medium under different temperatures for 2.5 days. (**B**,**C**) Quantitative total conidial production and colony diameter for the indicated strains shown in Panels A. (**D**) Colony morphology of the Δ*trpR* and reference strains on solid PDRUU medium supplemented with 5 mM CR, 20 mM CFW, or 0.1 μM CAS at 37 °C for 2.5 days. (**E**,**F**) Quantitative total conidial production and colony diameter for the indicated strains shown in Panels D. Values represent mean ± SD of three replicates. (ns, not significant; **, *p* < 0.001; ***, *p* < 0.001; ****, *p* < 0.0001).

To investigate the biological function of TrpR in *A. nidulans*, we constructed a null mutant carrying a deletion of the gene encoding TrpR (AN9146) by replacing the coding sequence with the *pyrG* selectable marker in the parental TN02A7 strain. The generation of the Δ*trpR* mutant was confirmed to be successful by a diagnostic PCR (see Figure S1B). As shown in Figure 3A–C, compared to the parental wild-type strain, removing the *trpR* did not affect the colony diameter on the PDRUU medium but the number of conidia in the Δ*trpR* mutant markedly reduced. Despite the fact that the parental wild type contained 100% of conidia, the Δ*trpR* mutant only reached 45% of WT at 30 °C and 10% of WT at 37 °C. Nevertheless, when cultured at 42 °C, the conidiation was almost abolished in Δ*trpR*. In comparison, the parental wild-type strain still displayed a robust conidia production at 42 °C, although WT also displayed relatively decreased conidia compared to its levels at 37 °C. To confirm that the defective phenotype was specifically caused by the *trpR* deletion, we constructed a *trpR* complemented strain by reintroducing *trpR* into the Δ*trpR* mutant. The results revealed that *trpR^c^* could resolve the defects of the Δ*trpR* mutant, suggesting that a lack of TrpR causes a marked reduction in conidial production, especially under thermal culture stresses (Figure 3).

Since the TRP channel superfamily in mammals and in yeasts performs critical functions in the response to external stimuli, and in the knowledge that its members are referred to as the “vanguards of the sensory system”, we postulated as to whether TrpR in *A. nidulans* possesses the ability to “sense” external stimuli. Thus, we compared the growth of Δ*trpR* and the parental wild-type strain in the presence of different stress agents (Figure S2). We found that Δ*trpR* was hypersensitive to cell wall stressors, including the chitin-binding agents calcofluor white (CFW) and congo red (CR), and the β-1,3-glucan synthase inhibitor caspofungin (CAS) compared to the parental wild-type strain, as shown in Figure 2D–F. Altogether, these results suggest that the Δ*trpR* mutant may have cell wall defects, and that a lack of TrpR results in a drastic reduction in the number of conidia produced in a high temperature-induced defect-dependent manner.

### 3.4. Defects of the ΔtrpR Mutant Can Be Rescued by Adding Extracellular Ca^2+^

**Figure 4:**
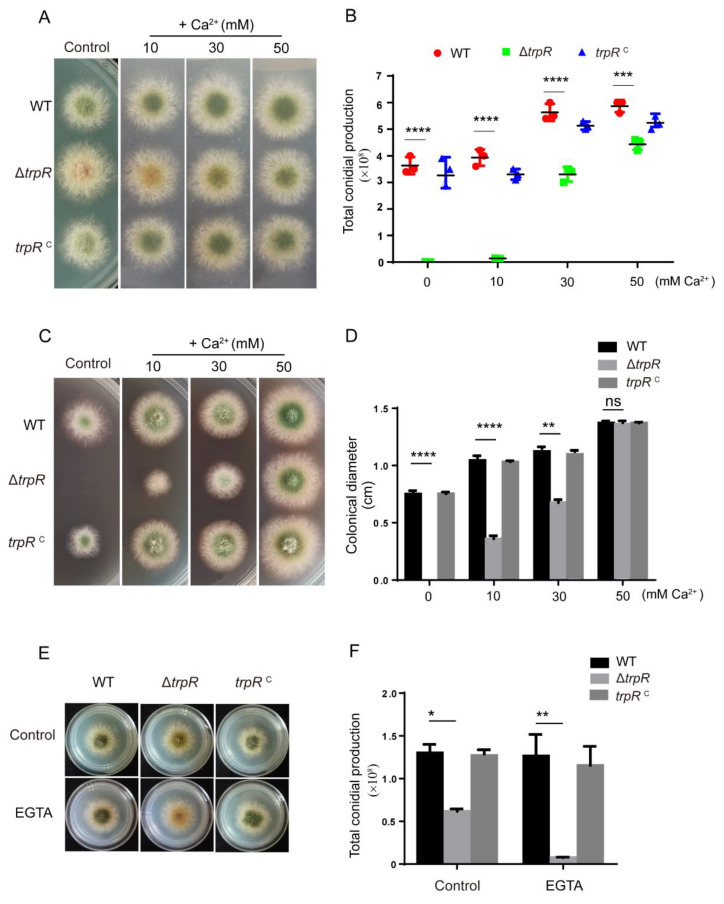
Sensitivity to thermal and cell wall stress agents in the Δ*trpR* mutant can be restored by adding calcium. (**A**) Colony morphology for the indicated strains grown on solid PDRUU medium in the absence or presence of 10, 30 and 50 mM CaCl_2_ at 37 °C for 2.5 days. (**B**) Quantitative total conidial production for the strains shown in Panel A. (**C**) Colony morphology for the indicated strains grown on solid PDRUU medium supplemented with 5 mM CR and in the absence or presence of 10, 30, 50 mM CaCl_2_ at 37 °C for 2.5 days. (**D**) Quantitative total colony diameter for the strains shown in Panel C. (**E**) Colony morphology for the indicated strains grown on solid PDRUU medium supplemented with 1.2 M sorbitol at 37 °C for 2.5 days. (**F**) Quantitative total conidial production for the strains shown in Panel E. Values represent mean ± SD from three replicates. (ns, not significant; *, *p* < 0.05; **, *p* < 0.001; ***, *p* < 0.001; ****, *p* < 0.0001).

Previous studies reported that TRP channels perform crucial roles in the regulation of cytoplasmic Ca^2+^ in fission yeast. To determine whether the defects mentioned above in *trpR* are related to intracellular calcium homeostasis, we added CaCl_2_ to MM and then compared the growth of Δ*trpR* and the parental wild-type strain. As shown in Figure 4A–D, we found that an addition of calcium was able to not only dramatically restore the conidiation in the Δ*trpR* mutant but was also able to alter the hypersensitivity of Δ*trpR* for the insensitive phenotype under treatment with cell wall stress agents, which displayed phenotypes similar to that of the parental wild type. In addition, the phenotypic restoration had a dose-dependent manner. After the addition of 50 mM Ca^2+^, the defective phenotypes of Δ*trpR* was almost restored to the level the of parental wild-type strain (see Figure 4C,D). In contrast, the addition of calcium chelator-EGTA exacerbated the conidiation defects in the Δ*trpR* mutant (Figure 4E,F).

To further test the specification of Ca^2+^, we added other divalent cations, including Mg^2+^ Cu^2+^, Co^2+^, Mn^2+^ at the indicated concentrations (see Figure S3E) into media and found that the addition of Mg^2+^ could also partly restore the defective phenotypes (see Figure S3A–D), but other ions were unable to rescue the defects of Δ*trpR*.

Taken together, these results suggest that TrpR is involved in the Ca^2+^ uptake when subject to low calcium conditions and that partially increasing the amount of extracellular calcium and Mg^2+^ can bypass the requirement of TrpR in *A. nidulans*.

### 3.5. Genetic and Functional Relationship between TrpR and the Previously Identified High-Affinity Calcium Channels CchA/MidA

**Figure 5:**
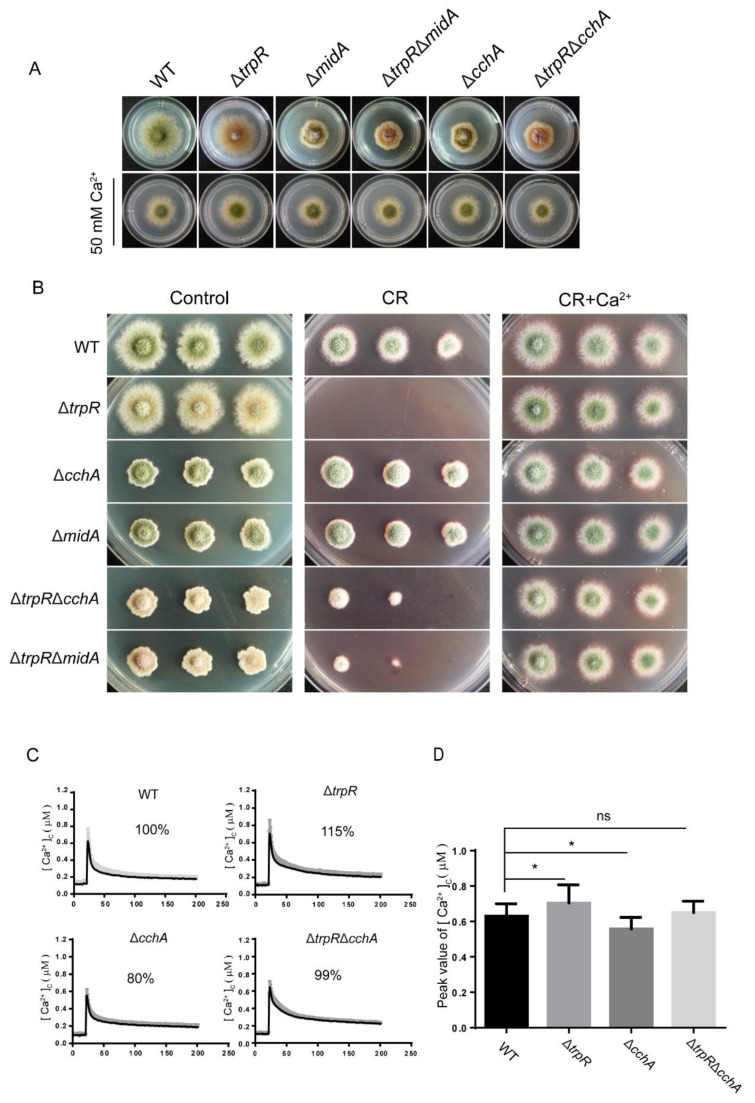
TrpR displays an opposite function with MidA/CchA. (**A**) Colony morphology for the indicated strains grown on a solid PDRUU medium in the presence or absence of 50 mM CaCl_2_ at 37 °C for 2.5 days. (**B**) Colony phenotypes of the indicated strains at a series of 2.5 μL 10-fold dilutions derived from a starting suspension of 10^6^ conidia·ml^−1^ grown on solid PDRUU medium supplementation with 5 mM CR and in the presence or absence of 50 mM CaCl_2_ at 37 °C for 2.5 days. (**C**) Real-time monitoring of the [Ca^2+^]c of the indicated strains following stimulation with 100 mM CaCl_2_. (**D**) Quantitative result of the peak of transient [Ca^2+^]c of the indicated strains shown in Panel C. Values represent mean ± SD from three replicates. (ns, not significant; *, *p* < 0.05).

Previous studies have identified that under calcium-limiting conditions, the high affinity calcium system (HACS)-CchA/MidA is required for colony growth. TrpR displayed a function similar to the function of MidA/CchA. We then wanted to investigate the relationship between the MidA/CchA complex and TrpR in regulating calcium homeostasis and adapting to cell wall stress. For the purpose of analyzing the genetic phenotype, we generated Δ*trpR*Δ*midA* and Δ*trpR*Δ*cchA* mutants via genetic crossing, as described in the Materials and Methods section. In our study, and consistent with previous reports, the Δ*midA* and Δ*cchA* mutants displayed a smaller colony size with a decreased level of conidium production compared to that of the parental wild-type strain when grown on a solid PDRUU medium. In comparison, the Δ*trpR*Δ*midA* and Δ*trpR*Δ*cchA* double mutants presented overlapping aggravated defects when compared with both Δ*trpR* and Δ*cchA* or Δ*midA* single mutants. (Figure 5A,B).

Furthermore, when the medium was supplemented with the cell wall destruction reagent-congo red (CR), the Δ*trpR* mutant became hypersensitive to CR and colony growth was not detectable. In contrast, the Δ*midA* and Δ*cchA* mutants were resistance to CR, displaying a colony diameter that is similar to the colony diameter of the parental wild-type strain. Interestingly, exogenous Ca^2+^ substantially overcame all the defects seen in the single or double mutants under either thermal or CR stress (Figure 5A,B). These data implied that TrpR may perform a function opposite to CchA/MidA under calcium-limiting conditions, while abundant calcium addition is able to bypass the requirement for three of them, which means that TrpR may also belong to a member of the high-affinity uptake system. Under high calcium concentrations, there are other uptake systems to regulate Ca^2+^ homeostasis in the absence of TrpR and CchA or MidA.

To test the functional relationship of TrpR with CchA/MidA, we monitored the extracellular calcium-induced cellular calcium transients [Ca^2+^]c in the living cells of *A. nidulans* using the expressed codon-optimized aequorin. When treated with 0.1 M CaCl_2_, the [Ca^2+^]c concentration in wild-type cells transiently increased from a resting level of approximately 0.1 μM to a peak concentration of 0.6 μM. In comparison, the Δ*trpR* mutant displayed a greater increase of 115% compared to the parental wild type (100%) in the [Ca^2+^]c amplitudes. In contrast, the Δ*cchA* mutant showed a reduction in the [Ca^2+^]c amplitude by approximately 80% as compared to the parental wild type under the same stimulating conditions. Notably, unlike the single mutant TrpR or CchA, the Δ*trpR*Δ*cchA* double mutant displayed a comparable [Ca^2+^]c amplitude to that of the parental wild-type strain (Figure 5C,D). These data suggest that TrpR and CchA have a reversed function in regulating cellular calcium transients [Ca^2+^]c, implying that the HACS components of MidA and CchA are required for the Ca^2+^ influx from the extracellular environment while TrpR is primarily responsible for the transport of calcium from the cytoplasm into the Golgi apparatus.

### 3.6. Lack of Golgi-Localized ATPase PmrA Severely Aggravates Defects of ΔtrpR

**Figure 6:**
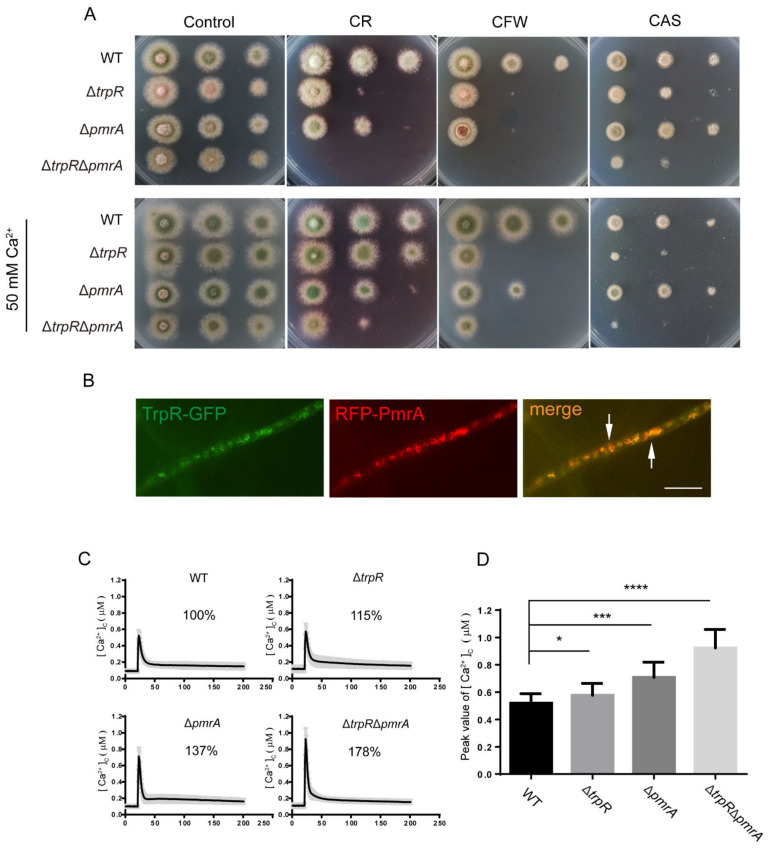

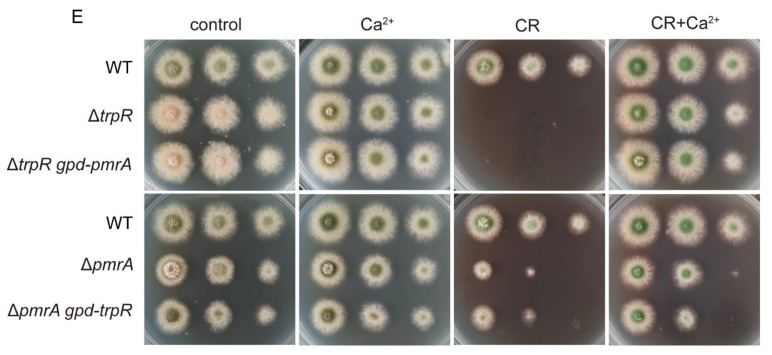
Loss of *pmrA* aggregates the defects of thermal/cell wall stress agent sensitivity in the Δ*trpR* mutant. (**A**) Colony phenotypes of the indicated strains at a series of 2.5 μL 10-fold dilutions derived from a starting suspension of 10^6^ conidia·ml^−1^ grown on solid PDRUU medium supplemented with 50 mM Ca^2+^ and in the presence or absence of 5 mM CR, 20 mM CFW, or 0.1 μM CAS at 37 °C for 2.5 days. (**B**) Distribution of TrpR-GFP relative to the RFP-PmrA. Overlapping positions are indicated with an arrow at the merged picture, Scale bar, 5 μm. (**C**) Real-time monitoring of the [Ca^2+^]c of the indicated strains following stimulation with 100 mM CaCl_2_. (**D**) Quantitative the peak of transient [Ca^2+^]c of the indicated strains shown in Panel C. Values represent mean ± SD from three replicates. (*, *p* < 0.05; ***, *p* < 0.001; ****, *p* < 0.0001). (**E**) Colony phenotypes of the indicated strains at a series of 2.5 μL 10-fold dilutions derived from a starting suspension of 10^6^ conidia·ml^−1^ grown on solid PDRUU medium supplemented with 5 mM CR and in the presence or absence of 50 mM Ca^2+^ at 37 °C for 2.5 days.

The P-type Golgi Ca^2+^ ATPase PmrA is an *A. nidulans* homolog of yeast Pmr1, which localizes at the Golgi and is responsible for the Ca^2+^ transport from the cytoplasm into the Golgi. In order to investigate the relationship between TrpR and PmrA, we generated Δ*pmrA* and Δ*trpR*Δ*pmrA* mutants. As shown in Figure 6A, the Δ*pmrA* mutant and the Δ*trpR*Δ*pmrA* double deletion mutant showed a slight reduction in the hyphal radial growth and conidiation compared to the wild-type strain on a minimal PDRUU media. However, for the treatment of the cell wall perturbation with congo red (CR) and calcofluor white (CFW), the Δ*trpR*Δ*pmrA* double deletion mutant did not show a colony sign (if any). Similarly, under the medium supplemented with the cell wall targeted antifungal CAS, Δ*trpR*Δ*pmrA* displayed very severe colony defects with tiny fluffy colonies when cultured at 37 °C. Supplemented Ca^2+^ in the medium was unable to rescue all defects in the Δ*trpR*Δ*pmrA* mutant under cell wall stress conditions. These data suggest that a lack of both TrpR and PmrA is cannot be accounted for by other Ca^2+^ uptake systems for cell wall stress tolerance in *A. nidulans* (Figure 6A).

To further assess whether TrpR is colocalized with Golgi-localized PmrA, we labeled PmrA with an RFP tag in the TrpR-GFP background. A microscopic examination showed that most of the TrpR colocalized with PmrA (Figure 6B). To further test the transient [Ca^2+^]c change in these mutants, the Δ*pmrA*, Δ*trpR* and Δ*trpR*Δ*pmrA* mutants were exposed to a 0.1 M CaCl_2_ stimulus, respectively, and the [Ca^2+^]c amplitude of Δ*trpR*Δ*pmrA* showed a remarkably unusual increase to approximately 178% compared to 100% of the parental wild-type strain. The single deletion mutant Δ*trpR* or Δ*pmrA* also resulted in a significantly higher [Ca^2+^]c amplitude compared to the parental wild type but less than that of the double deletions of Δ*trpR*Δ*pmrA,* indicating that the perturbation of calcium homeostasis induced by the *trpR* deletion could be further aggravated by a loss of *pmrA* (Figure 6C,D), and that TrpR may have a parallel function with PmrA in transporting the cellular [Ca^2+^]c into the Golgi in response to extracellular calcium.

Given that PmrA and TrpR perform similar functions, we next wondered whether the defective phenotype caused by *trpR* deletion could be compensated for by an overexpressed *pmrA* and *vice versa*. Strains were overexpressed via the introduction of *trpR* and *pmrA* under the control of a constitutive promoter gpd into the Δ*pmrA* and Δ*trpR* backgrounds, respectively. The RT-PCR verified that the expression of *trpR* in the Δ*pmrA gpd-trpR* strain was approximately 8 times higher than that of the parental wild-type strain, and *pmrA* in the Δ*trpR gpd-pmrA* strain was nearly 20 times higher than that of the wild-type strain. (See Figure S4). These data confirmed that the *gpd* promoter induced a high expression for its controlled genes at the mRNA level. However, as shown in Figure 6E, there was almost no difference between the mutant strains and the corresponding overexpressing strains. These results may imply that, although TrpR and PmrA had similar effects in regulating [Ca^2+^]c, they still had independent characteristic functions, and a compensatory relationship probably did not exist between TrpR and PmrA.

### 3.7. TrpR Is Involved in the Normal Cell Wall Architecture and Composition

**Figure 7:**
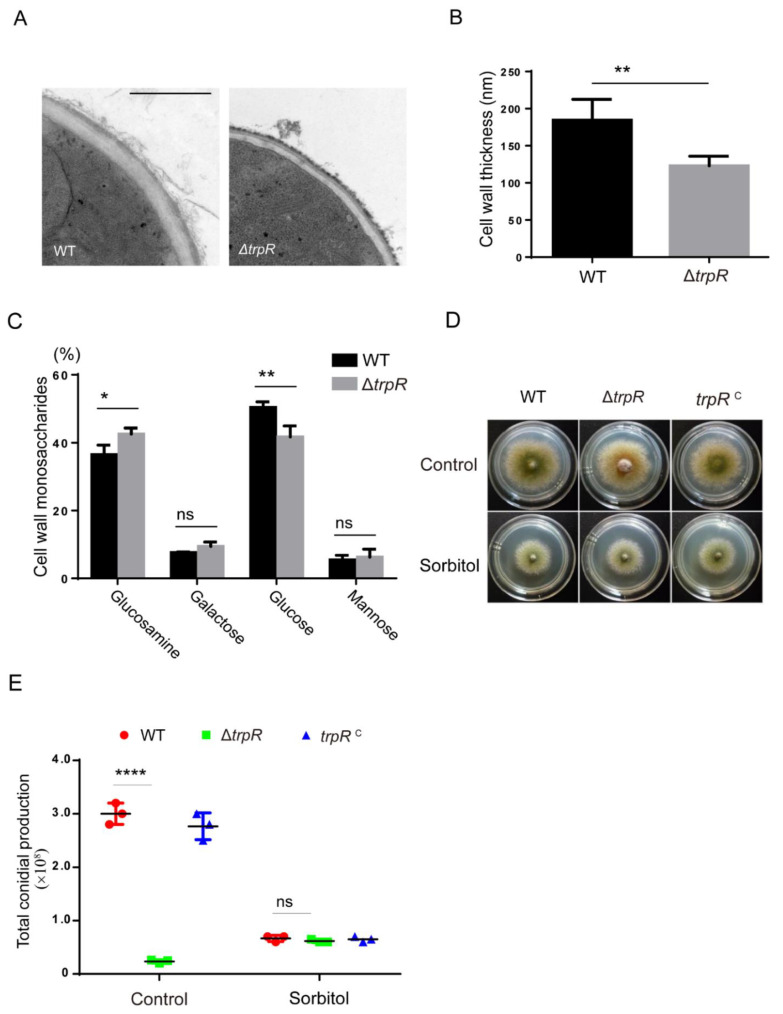
TrpR regulates cell wall architecture and compositions. (**A**) Representative TEM images of the hyphae of WT and Δ*trpR* strains cultured in a liquid PDRUU medium. Scale bar, 100 nm. (**B**) Quantification of the mean cell wall thickness of WT and Δ*trpR* strains as in Panel A. The values represent the mean ± SD from three replicates, with at least 10 sections measured for each strain. (**C**) Complete monosaccharide composition of WT and Δ*trpR* mutant mycelial cell walls. (**D**) Colony morphology for the indicated strains grown on solid PDRUU medium in the presence of 1.2 M sorbitol at 37 °C for 2.5 days. (**E**) Quantitative total conidial production for the strains shown in Panel D. Values represent mean ± SD from three replicates. (ns, not significant; *, *p* < 0.05; **, *p* < 0.01; ****, *p* < 0.0001).

Given that the Δ*trpR* mutant was hypersensitive to cell wall perturbation stress reagents, a lack of TrpR may affect cell wall formation. To explore the effects of TrpR on cell wall architecture, the hyphal cell wall was inspected using transmission electron microscopy (TEM). The results revealed a significantly thinner cell wall in the Δ*trpR* mutant than in the wild-type strain, indicating that TrpR plays a pivotal role in cell wall architecture (Figure 7A,B). In addition, we further analyzed the cell wall monosaccharide compositions in mutants and wild-type strains using high-performance ion chromatography. The results revealed abnormalities in the Δ*trpR* mutant compared to the parental strain (Figure 7C). The loss of TrpR made an impact on cell wall composition, resulting in an increased proportion of glucosamine but a decreased level of glucose in the cell wall compared with the wild-type strain. To further test whether colony defects of Δ*trpR* result from cell wall dysfunction, we added sorbitol, an osmotic stabilizer to recover the phenotype of a fragile cell wall. As shown in Figure 7D,E, although sorbitol was able to facilitate a remarkably reduced conidiation for the wild-type strain, the Δ*trpR* mutant evidenced clear recovery of conidial production through the treatment of sorbitol compared to that which was observed using the minimal medium with the absence of sorbitol. Consequently, there was no significant difference for conidial number between Δ*trpR* and its parental wild type or between Δ*trpR* and the *trpR* complementary strain in the presence of sorbitol which suggest that the colony defects of Δ*trpR* may be a result of the cell wall dysfunction (Figure 7D,E). Thus, these data suggest that TrpR plays a crucial role in cell wall composition and architecture and that the osmotic stabilizer is capable of rescuing the conidial defect for deletion of *trpR*.

## 4. Discussion

TRP proteins constitute a superfamily that encodes transmembrane ion channels with very diverse permeation and gating properties [2,3,4]. In mammals, TRP channels are best known as sensors for environmental irritants inducing somatosensory responses [33,34]. Members of the TRP family are intended to be conserved from fungi to mammals. However, the biological functions of TRP channels are not defined in filamentous fungi. In this study, we aimed to find some putative TRP proteins in filamentous fungus *Aspergillus nidulans* by using TRP Pfam (PF06011) as one of the search criteria in the hidden Markov Model analysis (hmmscan). According to a bioinformatics analysis, we found 4 TRP domain-containing proteins that are putative members of TRP channels in filamentous fungus *A. nidulans*. Among them, deleting AN9146, which is referred to as TrpR, displayed colony defects especially under thermal, calcium-limited and cell-wall-stress cultural conditions. Through a phenotypic comparison using gene deletion, overexpression and mutant crossing techniques, we demonstrated that the putative transient receptor potential protein TrpR, a major calcium transporter, is involved in asexual conidiation and the response to cell wall stress adaption by affecting cellular calcium regulation in the filamentous fungus *A. nidulans*.

In mammals, much evidence has demonstrated that TRP channels perform important roles in physiological and pathological processes, and the protein structure and the specific inhibitor of some TRP members have been recognized to some extent [2]. TRP homologs were also identified [33,34] in yeast. The first TRP protein found in *S. cerevisiae* is TRPY1, is located in vacuoles and is responsible for the modulation of cytosolic calcium signaling by releasing Ca^2+^ from the vacuole in response to hyperosmotic stress and its activation and inhibition are modulated by calcium and lipid, respectively [12,14]. However, the current analysis shows that TRPY1 is a member of V-type of the TRP cation channel subfamily (Interpro entry IPR024862) and that there is no TRP Pfam domain inside of TRPY1′s protein sequence, based on the protein information resource database InterPro (http://www.ebi.ac.uk/interpro/, accessed on 23 October 2021). We therefore could not find a TRPY1 homolog in *A. nidulans* by searching the TRP Pfam (PF06011). Besides, the three TRP-like ion channels were identified in *S. pombe*: *pkd2*, *trp1322*, and *trp663*. The deletion of either Trp1322 or Pkd2 lowers the ability to maintain intracellular Ca^2+^ homeostasis [13]. The Pkd2 channel was also found to be involved in cell wall synthesis, membrane proteins transport and cytokinesis [15,16]. In this study, we found that most fungal homologs of TrpR display signatures typical of the TRP_N (PF02221) domain and TRP Pfam (PF06011), which are relatively conserved in various fungi. Previous studies identified that the TRP_N (or called ML) domain might be involved in mediating diverse biological functions through an interaction with specific lipids [35,36,37], which means that TRP homologs may need to recognize lipids in order to function properly.

Our findings in this study indicate that TrpR possesses dynamic cellular localizations at the internal membrane system and septa and a weak localization at the plasma membrane of germlings, while the majority of TrpR is highly localized at the membranes of the Golgi and vesicles. These data indicate that TrpR in *A. nidulans* may perform important functions during the different developmental stages. Notably, a lack of TrpR caused a marked conidiation reduction, and the conidiation in Δ*trpR* was almost nonexistent at 42 °C. In comparison, the deletion of *trpR* did not affect the colony size, suggesting that TrpR is more often required for asexual reproduction than for hyphal growth. In addition, we noticed that the *trpR* mutant was hypersensitive to cell wall destruction reagents (CR, CFW and an antifungal CAS), implying that cell wall integration requires the function of TrpR. Interestingly, these defective phenotypes could be completely resolved via the addition of extracellular calcium. In contrast, the defects in the Δ*trpR* mutant were exacerbated by adding the calcium chelator-EGTA. These data suggest that TrpR is calcium permeable and may affect cell wall integrity by regulating calcium homeostasis.

A crosstalk between the calcium signal pathway and cell wall integrity pathway has been reported in yeast and some filamentous fungi. Many studies have demonstrated that losses of function in the mutants of calcium regulators could lead to defects of the cell wall integrity [38]. Findings in this study suggest that cell wall defects that were induced by deletion of TrpR may be due to two possibilities. The first is the abnormal expression of cell wall synthetase as regulated by cellular calcium homeostasis in TrpR mutants since previous studies have reported that in *A. fumigatus*, a major calcium-related transcription factor CrzA was able to regulate the expression of chitin synthases by binding to the calcineurin-dependent response elements (CDRE) in their promoter [39,40]. The second possibility may be a result of the imbalance of calcium signaling in Golgi apparatus in Δ*trpR* results in abnormal cell-wall integrity since cell wall material transport requires the normal functioning of the Golgi apparatus [25].

In addition, the defects in the Δ*trpR* mutant could also be rescued by the addition of Mg^2+^, which suggests that TrpR may be a nonspecific transmembrane ion channel involving calcium and magnesium ion transportation, similar to that in mammalian TRP proteins. In human cells, the TRPM subfamily contains similar characteristic that can be permeable to calcium and magnesium ions [41]. Among the members of the TRPM subfamily, TRPM2 was found to be permeable to Ca^2+^, Mg^2+^, and monovalent cations [2]. TRPM6 and TRPM7 are most often studied with regard to their roles in mammals and cellular Mg^2+^ homeostasis [42]. These data suggested that the TrpR may be a relative of the TRPM subfamily.

Moreover, the functional and genetic relationship between TrpR and the known calcium channels CchA/MidA was determined by monitoring the cellular calcium transients and colony phenotypes under calcium-limited culture conditions [22,23], indicating that TrpR may also belong to a member of the high-affinity Ca^2+^ influx system, but TrpR seems to perform a function opposite to the function of the CchA/MidA complex in regulating cellular calcium transients since the double mutant Δ*trpR* Δ*cchA* displayed a recovery of the normal calcium transient compared to the calcium transient of the single mutant, resulting in a calcium response peak similar to that in the parental wild-type strain. In contrast, the double deletion of both TrpR and PmrA displayed an overlaid abnormal increase in calcium transients and hypersensitivity to cell wall destruction reagents. Nevertheless, the colocalization at the membrane of the Golgi for TrpR and PmrA suggested that these two channels may work as ion transporters, transferring Ca^2+^ from the cytosol into the Golgi apparatus and maintaining cellular calcium homeostasis [24]. However, an overexpressed *trpR* in Δ*pmrA* or vice versa was unable to recover defects for the single mutation of *trpR* or *pmrA*, suggesting that TrpR might contain a parallel but irreplaceable function with that of P-type Golgi Ca^2+^ ATPase-PmrA. However, we could not overlook the possibility that overexpressed strains may not induce normal functions at the protein level, since we only verified the overexpressed strains at the mRNA level. Furthermore, future work may provide insight in the relationship between TrpR with PmrA at the protein level.

Taken together, the data in this study demonstrated a bioinformatics predicted TRP ion channel, TrpR, which performs an important role in conidiation and cell wall integration by transferring Ca^2+^ from the cytosol into the Golgi apparatus. Calcium homeostasis, which is maintained by TrpR, is required for cell wall integration and for the response to thermal and cell wall stresses.

## Figures and Tables

**Table 1:** All *A. nidulans* strains used in this study.

Strains	Genotype	Source
TN02A7	*pyrG89; pyroA4; nkuA::argB2; riboB2; veA1*	FGSC
TrpR-GFP	*pyrG89; pyroA4; nkuA::argB2; trpR::gfp-pyrG; riboB2; veA1*	This study
TrpR-GFP mRFP-PH^OSBP^	*pyrG89; pyroA4; nkuA::argB2; trpR::gfp-pyrG; riboB2; veA1;* *gpd-* *mRFP-* *PH^OSBP^;*	This study
TrpR-GFP RFP-PmrA	*pyrG89; pyroA4; nkuA::argB2; trpR::gfp-pyrG;* *gpd::* *RFP::* *PmrA;* *riboB2; veA1;*	This study
Δ*trpR*	*pyrG89; pyroA4; nkuA::argB2;* Δ*trpR::pyrG; riboB2; veA1*	This study
Δ*trpR::pyroA*	*pyrG89; pyroA4; nkuA::argB2;* Δ*trpR::pyroA; riboB2; veA1*	This study
Δ*cchA*	*pyrG89; pyroA4; nkuA::argB2;* Δ*cchA::pyrG; riboB2; veA1*	[21]
Δ*midA*	*pyrG89; pyroA4; nkuA::argB2;* Δ*midA::pyrG; riboB2; veA1*	[21]
Δ*pmrA*	*pyrG89; pyroA4; nkuA::argB2;* Δ*pmrA::pyroA; riboB2; veA1*	[24]
Δ*trpR*Δ*cchA*	*pyrG89; pyroA4; nkuA::argB2;* Δ*trpR::pyrG;* Δ*cchA::pyroA; riboB2; veA1*	This study
Δ*trpR*Δ*midA*	*pyrG89; pyroA4; nkuA::argB2;* Δ*trpR::pyroA;* Δ*midA::pyrG; riboB2; veA1*	This study
TN02A7-AEQ	*pyrG89; pyroA4; nkuA::argB2; veA1;* pAEQ	This study
Δ*trpR*-AEQ	*pyrG89; pyroA4;* Δ*trpR::pyrG; nkuA::argB2; veA1;* pAEQ	This study
Δ*cchA*-AEQ	*pyrG89; pyroA4;* Δ*cchA::pyrG; nkuA::argB2; veA1;* pAEQ	This study
Δ*trpR*Δ*cchA*-AEQ	*pyrG89; pyroA4;* Δ*trpR::pyroA;* Δ*cchA::pyrG; nkuA::argB2; veA1;* pAEQ	This study
Δ*pmrA*-AEQ	*pyrG89; pyroA4;* Δ*pmrA::pyrG; nkuA::argB2; veA1;* pAEQ	This study
Δ*trpR*Δ*pmrA*-AEQ	*pyrG89; pyroA4;* Δ*trpR::pyroA;* Δ*pmrA::pyrG; nkuA::argB2; veA1;* pAEQ	This study
Δ*pmrA gpd-trpR*	*pyrG89; pyroA4;* Δ*pmrA::pyroA; pyrG-gpd-trpR; nkuA::argB2; veA1;*	This study
Δ*trpR gpd-pmrA*	*pyrG89; pyroA4;* Δ*trpR::pyroA; pyrG-gpd-pmrA; nkuA::argB2; veA1;*	This study

**Table 2:** Primers used in this study.

Primer Names	Sequence 5′ to 3′
trpR P1	AGGGCGGTTGTTGATACGCT
trpR P3	AAGAGCATTGTTTGAGGCCGCGACCTTTCTATATGAATG
trpR P4	CTTGGCATCACGCATCAGTAGGCTCTCGCATTTCTTC
trpR P6	CATTTCGGCTCTACTGCTC
trpR P2	CGGTCGCCTACTGATTCTC
trpR P5	GGCGTAGACGGTGGGAAAT
trpR diag F	TAGCATTCCGACCCTTCCC
trpR diag R	GCCTTTCCCTTTATCCTTTG
pyrG diag F	AGAGTATGCGGCAAGTC
pyrG diag R	AAACCAGAAGAAACCTCCC
trpR^c^ up	CTGTTACTGAGCGGTTCTGAG
trpR^c^ down	GATGACATCTTGGCGCTGGTAG
trpR-gfp P1	GAGAGAGATCCCACAGCCGA
trpR-gfp P3	CCAGCGCCTGCACCAGCTCCTGTGTATCGTGAGGAAGC
trpR-gfp P4	TCTGAGAGACGAATTGGCATTAGGCTCTCGCATTTCTTC
trpR-gfp P6	CATTTCGGCTCTACTGCTC
trpR-gfp P2	TAGACCTCGTAAACGGACTG
trpR-gfp P5	GGCGTAGACGGTGGGAAAT
gfp diag R	CATTGAACACCATAGGTGA
PHosbp F	GCATGCGGAGAGACGGACG
PHosbp R	TCACGAATTCTTCTTCACAGC
gpd-rfp-pmrA up	CCTTTAATCAAGCTTATCGATATGAGAAATGTCAAGGTCCGC
gpd-rfp-pmrA down	CTCGAGGTCGACGGTATCGATCAGAGGCACTCTTCCGACCAGT
gfp up	GGAGCTGGTGCAGGCGCTGG
pyrG down	GCCTCAAACAATGCTCTTCA
gpd up	CCTTTAATCAAGCTTATCGAT
gpd down	CTCGAGGTCGACGGTATCGAT
gpd-trpR up	CCTTTAATCAAGCTTATCGATATGGATTTCGATTACAACGCGA
gpd-trpR down	CTCGAGGTCGACGGTATCGATTCATGTGTATCGTGAGGAAG
gpd-pmrA up	CCTTTAATCAAGCTTATCGATATGAGAAATGTCAAGGTCCGC
gpd-pmrA down	CTCGAGGTCGACGGTATCGATTTACACATTCACGCTATATCC
Aeq F	ATGACCTCCAAGCAGTAC
Aeq R	TTAGGGGACGGCACCGCCGTA
RT-tub F	GCCGGTATGGGTACTCTTTTG
RT-tub R	GTCTCATCGGAGTGCTCAACG
RT-trpR F	TGATGACCTACCCACCGTACTCTT
RT-trpR R	GGCTATTCCTGACGCTTGAACT
RT-pmrA F	GGCTTCGATTCCTCAAACAG
RT-pmrA R	AAACCCAACCGTAACCACAA

## Data Availability

All data are publicly available.

## Supplementary Materials

The following are available online at https://www.mdpi.com/article/10.3390/jof7110920/s1, Figure S1: Diagnostic PCR analyses of the indicated strains, Figure S2: Δ*trpR* mutant showed no hypersensitivity to various stress agents, Figure S3: The defects in the Δ*trpR* mutant can be restored by excess magnesium, Figure S4: Verification of the overexpression strains.
