# Supplementary material for: A Transient Receptor Potential-like Calcium Ion Channel in the Filamentous Fungus Aspergillus nidulans
Source: J Fungi (Basel). 2021 Oct 28;7(11):920. doi: 10.3390/jof7110920 (PMC8618638; doi:10.3390/jof7110920)
Supplement: Supplementary file 1 [file jof-07-00920-s001.zip › jof-1426166-supplementary.pdf]

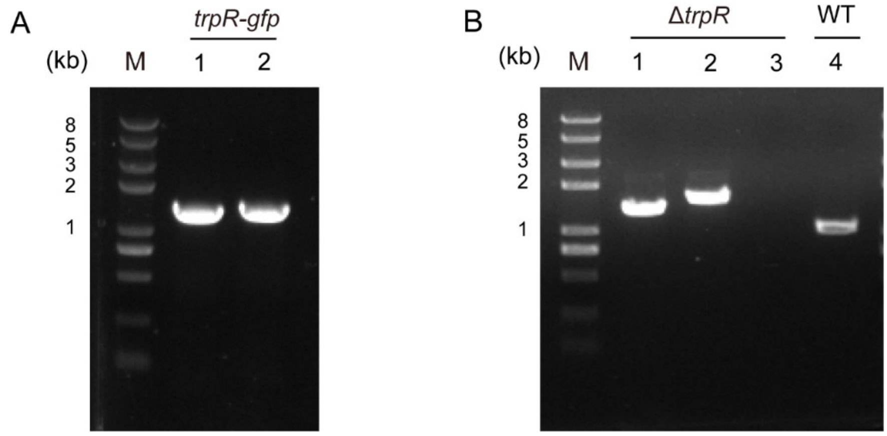

Figure S1. Diagnostic PCR analyses of the indicated strains. (A) The TrpR-GFP genomic DNA was used as template for diagnostic PCR with primer pairs trpR-gfp P1/gfp R for line 1, and pyrG F/trpR-gfp P6 for line 2. (B) As for the Δ*trpR* mutant, line 1 and line 2 were performed with primer pair trpR P1/ pyrG R and pyrG F/ trpR P6, respectively. Line 3 was performed with primer pair trpR diag F/trpR diag R suggesting the successful deletion for *trpR*. Line 4 showed the PCR products with primer pair trpR diag F/ trpR diag R using wild-type genomic DNA as template.

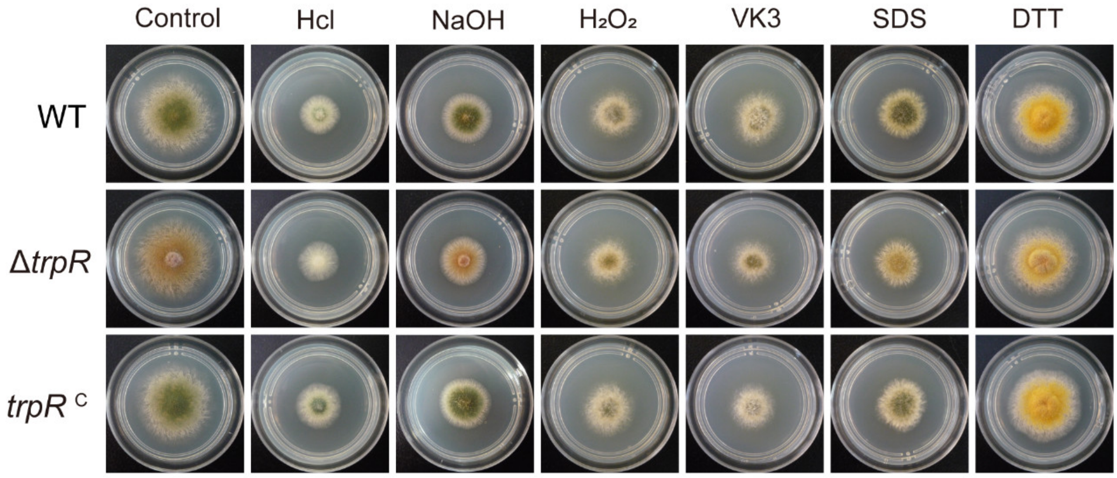

Figure S2. Δ*trpR* mutant showed no hypersensitivity to various stress agents. Colony morphology for the indicated strains grown on solid PDRUU in the absence or presence of 100 mM Hcl, 10 mM NaOH, 0.5 mM H_2O_2, 10 μM vitaminK3 (VK3), 0.005% SDS or 3 mM DTT at 37 °C for 2.5 days.

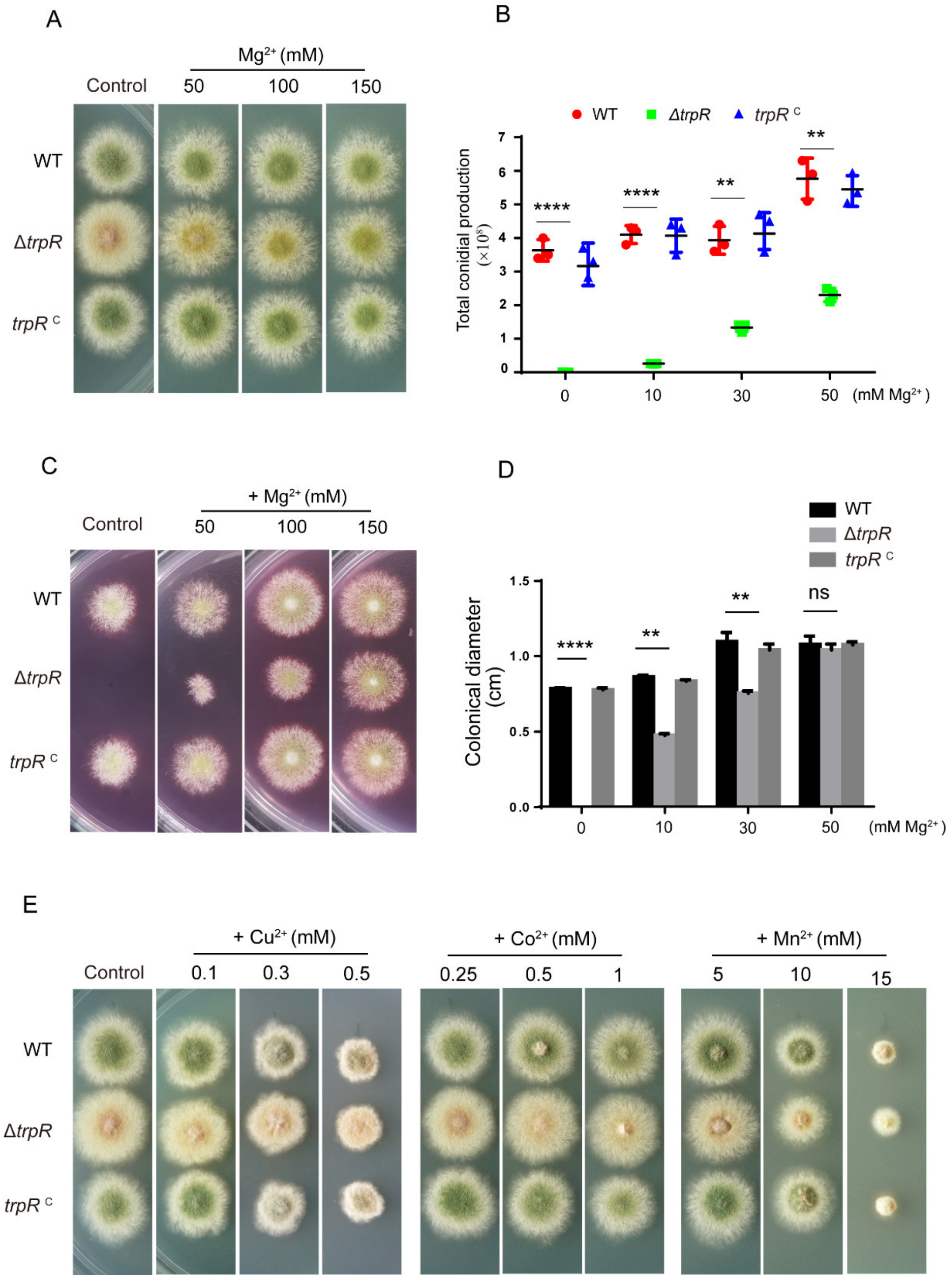

Figure S3. The defects in the Δ*trpR* mutant can be restored by excess magnesium. (A) Colony morphology for the indicated strains grown on solid PDRUU in the absence or presence of 50, 100 and 150 mM $MgCl_2$ at 37 °C for 2.5 days. (B) Quantitative total conidial production for the strains shown in panel A. (C) Colony morphology for the indicated strains grown on solid PDRUU supplemented with 5 μg/ml CR and in the absence or presence of 50, 100, 150 mM $MgCl_2$ at 37 °C for 2.5 days. (D) Quantitative total colonical diameter for the strains shown in panel C. Values represent mean ± SD from three replicates. (ns, not significant; **, P < 0.001; ***, P < 0.001; ****, P < 0.0001). (E) Colony morphology for the indicated strains grown on solid PDRUU supplemented with Cu^{2+}, Co^{2+} or Mn^{2+}.

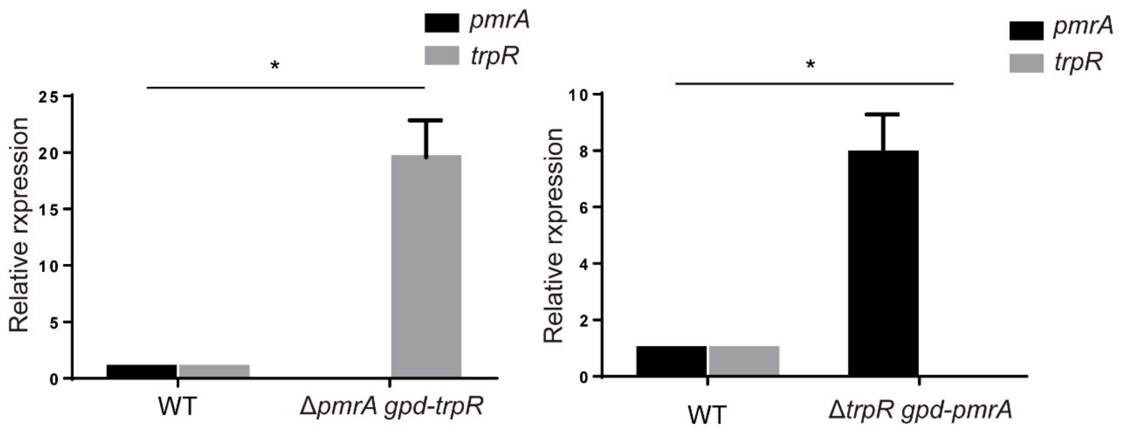

Figure S4. Verification of the overexpression strains. qRT-PCR analysis was performed to verify the Δ*pmrA gpd-trpR* (left) and Δ*trpR gpd-pmrA* (right) strains after growth of cultures in liquid PDR or PDRUU medium for 18 h at 37 °C. The *tubA* gene was used as an internal control. Values represent mean ± SD from three replicates. (*, $P < 0.05$).
